# Divergent Effects of EZH1 and EZH2 Protein Expression on the Prognosis of Patients with T-Cell Lymphomas

**DOI:** 10.3390/biomedicines9121842

**Published:** 2021-12-05

**Authors:** Franziska Lea Schümann, Elisabeth Groß, Marcus Bauer, Christian Rohde, Sarah Sandmann, Denis Terziev, Lutz P. Müller, Guido Posern, Andreas Wienke, Falko Fend, Martin-Leo Hansmann, Wolfram Klapper, Andreas Rosenwald, Harald Stein, Martin Dugas, Carsten Müller-Tidow, Claudia Wickenhauser, Mascha Binder, Thomas Weber

**Affiliations:** 1 Department of Internal Medicine IV, Hematology and Oncology, University Hospital Halle (Saale), Martin-Luther-University Halle-Wittenberg, 06120 Halle, Germany; franziska.schuemann@uk-halle.de (F.L.S.); elisabeth.gross@uk-halle.de (E.G.); denis.terziev@uk-halle.de (D.T.); lutz.mueller@uk-halle.de (L.P.M.); mascha.binder@uk-halle.de (M.B.); 2 Institute of Pathology, University Hospital Halle (Saale), Martin-Luther-University Halle-Wittenberg, 06112 Halle, Germany; marcus.bauer@uk-halle.de (M.B.); claudia.wickenhauser@uk-halle.de (C.W.); 3 Department of Medicine V, Hematology, Oncology and Rheumatology, University Hospital Heidelberg, 69120 Heidelberg, Germany; christian.rohde@med.uni-heidelberg.de (C.R.); carsten.mueller-tidow@med.uni-heidelberg.de (C.M.-T.); 4 Institute of Medical Informatics, University of Münster, 48149 Münster, Germany; sarah.sandmann@uni-muenster.de; 5 Institute for Physiological Chemistry, Medical Faculty, Martin Luther University Halle-Wittenberg, 06114 Halle, Germany; guido.posern@medizin.uni-halle.de; 6 Institute of Medical Epidemiology, Biometrics and Informatics, Martin-Luther-University Halle-Wittenberg, 06112 Halle, Germany; andreas.wienke@uk-halle.de; 7 Institute of Pathology, University Hospital Tübingen, 72076 Tübingen, Germany; falko.fend@medizin.uni-tuebingen.de; 8 Institute of Pathology and Molecular Pathology, Helios University Hospital Wuppertal, 42283 Wuppertal, Germany; m.l.hansmann@em.uni-frankfurt.de; 9 Department of Pathology, Hematopathology Section and Lymph Node Registry, University Hospital Schleswig-Holstein, Christian-Albrechts-University, 24105 Kiel, Germany; wolfram.klapper@uksh.de; 10 Institute of Pathology, University Würzburg, 97080 Würzburg, Germany; rosenwald@uni-wuerzburg.de; 11 Pathodiagnostik Berlin, 12099 Berlin, Germany; h.stein@pathodiagnostik.de; 12 Institute for Medical Informatics, University Hospital Heidelberg, 69120 Heidelberg, Germany; martin.dugas@med.uni-heidelberg.de

**Keywords:** T-cell non-Hodgkin’s lymphomas, PTCL, epigenetics, EZH1, EZH2, H3K27me3, immunohistochemistry, next generation sequencing

## Abstract

T-cell lymphomas are highly heterogeneous and their prognosis is poor under the currently available therapies. Enhancers of zeste homologue 1 and 2 (EZH1/2) are histone H3 lysine-27 trimethyltransferases (H3K27me3). Despite the rapid development of new drugs inhibiting EZH2 and/or EZH1, the molecular interplay of these proteins and the impact on disease progression and prognosis of patients with T-cell lymphomas remains insufficiently understood. In this study, *EZH1/2* mutation status was evaluated in 33 monomorphic epitheliotropic intestinal T-cell lymphomas by next generation sequencing and EZH1/2 and H3K27me3 protein expression levels were detected by immunohistochemistry in 46 T-cell lymphomas. Correlations with clinicopathologic features were analyzed and survival curves generated. No *EZH1* mutations and one (3%) *EZH2* missense mutation were identified. In univariable analysis, high EZH1 expression was associated with an improved overall survival (OS) and progression-free survival (PFS) whereas high EZH2 and H3K27me3 expression were associated with poorer OS and PFS. Multivariable analysis revealed EZH1 (hazard ratio (HR) = 0.183; 95% confidence interval (CI): 0.044–0.767; *p* = 0.020;) and EZH2 (HR = 8.245; 95% CI: 1.898–35.826; *p* = 0.005) to be independent, divergent prognostic markers for OS. In conclusion, EZH1/2 protein expression had opposing effects on the prognosis of T-cell lymphoma patients.

## 1. Introduction

Lymphoid neoplasms with T-cell differentiation are a heterogeneous group of rare diseases that are classified by the World Health Organization (WHO) in immature acute T-cell lymphoblastic leukemias (T-ALL) and mature post-thymic T-/NK-cell neoplasms (T-NHL) [1]. The latter, also known as peripheral T-cell lymphomas (PTCL), are classified into primary cutaneous, primary leukemic, and aggressive with nodal or extranodal manifestation, depending on the clinical presentation. For decades, the first-line standard therapy for most T-NHL subtypes has not changed. CHO(E)P (cyclophosphamide, doxorubicin, vincristine, (etoposide), and prednisone), a standard chemotherapy for B-cell lymphomas, is usually chosen as first-line treatment, although refractoriness and relapse are common [2,3].

Understanding of epigenetics in cancer development and tumor progression has improved rapidly in recent years, leading to the development of targeted therapies. Enhancer of zeste homologue 1 and 2 (EZH1/2), the catalytic subunit of polycomb repression complex 2 (PRC2), is one of the best studied histone-modifying enzymes and thus the target of numerous new therapeutics. EZH1/2 contains a catalytic domain at the COOH terminus that trimethylates the 27th lysine residue of histone H3 (H3K27me3) [4,5,6,7]. H3K27me3 is widely known as a marker for transcriptional repression [6,7,8]. The paralogues EZH1 and EZH2 have different expression patterns: while EZH1 is present in dividing and differentiated cells, EZH2 is found only in highly proliferative cells [9]. Furthermore, PRC2 complexes containing EZH2 have higher methyltransferase activity than those containing EZH1 [9]. Assuming that EZH1 compensates for the loss of EZH2 [10,11], not only the effect of EZH2-selective inhibitors, but also dual EZH1/2 inhibitors are currently under intense clinical investigation [12]. 

The basis for these new developments was the detection of *EZH1/2* alterations and/or EZH1/2 overexpression in many different neoplasms and their association with metastasis, poor prognosis, and treatment failure [13,14,15,16]. Mutations of *EZH1* and/or *EZH2* are described as rare or absent in T-cell lymphomas by a number of groups [17,18,19,20,21,22,23,24]. Furthermore, EZH1 expression has been reported to be low in T-NHL [25]. Even less is known about its association with progression or prognosis. In contrast, EZH2 is frequently overexpressed in T-NHL [25,26,27,28,29,30] and additionally appears to be associated with an unfavorable prognosis [30]. Several studies have shown associations between elevated EZH2 protein expression and various clinical markers such as the presence of B symptoms, elevated serum lactate dehydrogenase (LDH) levels, elevated ß2-microglobulin, and an increase in the proliferation marker Ki-67 [25,29,30,31]. 

In contrast to EZH2, several studies have demonstrated an inconsistent correlation between histone lysine modification H3K27me3 and cancer prognosis [32,33,34]. The conflicting results may suggest that methylation of different target genes occurs depending on its cellular context [35]. In patients with extranodal natural killer/T-cell lymphoma, nasal type (NKTCL), high H3K27me3 protein expression was associated with better prognosis and low clinical stages [31]. To date, expression in further subtypes of T-cell neoplasms has been studied less. A correlation between EZH2 and H3K27me3 in T-cell neoplasms was previously investigated by some research groups with conflicting results [26,31]. Thus, a possible non-canonical function (H3K27-specific histone methyltransferase) of EZH2 in oncogenesis is currently discussed [28,31]. 

Overall, T-cell lymphomas show a heterogeneous and so far insufficiently characterized profile of EZH1, EZH2, and H3K27me3 expression as well as a poorly studied correlation of these proteins with each other and with clinicopathological markers. To improve our understanding of these epigenetic factors, the present study evaluated the *EZH1/2* mutation status in a cohort of 33 monomorphic epitheliotropic intestinal T-cell lymphomas (MEITL) by next generation targeted sequencing and detected EZH1/2 and H3K27me3 expression levels by immunohistochemistry in 46 T-cell lymphomas. 

## 2. Materials and Methods

### 2.1. Patients and Samples

Thirty-three MEITL samples, classified by hematopathologists, were collected. While *EZH1* and *EZH2* mutation analysis by WES was performed in 11 samples, the mutation status of *EZH2* was analyzed in all 33 samples by targeted sequencing. Available patient characteristics are summarized in Table 1, but no survival data or further clinical data were available for these cases, so they were not included in further analyses of clinical and survival data.

A cohort of 46 patients with T-NHL and available survival data who received treatment at University Hospital Halle (Saale) between 2006 and March 2020 met inclusion criteria for tissue microarray (TMA) construction. T-NHL tissue samples were provided for TMA construction by the Institute of Pathology of the University Hospital Halle (Saale). The patients were identified through a review of the internal hospital database and those with available formalin fixated paraffin embedded (FFPE) T-cell lymphoma tissue were included in the sense of a convenience sample. Patients with an age below 18 years at initial diagnosis were excluded. The diagnosis of all patients who met the inclusion criteria was verified by two pathologists (C.W. and M.B. (Marcus Bauer)) according to the 2017 WHO criteria [1]. Five out of 57 original samples had to be excluded because the integrated tissue samples were not from the primary diagnosis or the diagnosis was not confirmed. Clinicopathological characteristics at the time of primary diagnosis including age, sex, histologic phenotype, B symptoms, Ann Arbor stage, International Prognostic Index (IPI), Eastern Cooperative Oncology Group (ECOG) status, bone marrow involvement (BMI), LDH level, white blood cell (WBC) count, Ki-67 expression, (response to) first-line chemotherapy, occurrence of relapses, and follow-up data were recorded. The study included 19 cases of peripheral T-cell lymphomas with T-helper phenotype (angioimmunoblastic T-cell lymphoma (AITL) and nodal peripheral T-cell lymphoma with T follicular helper phenotype (PTCL-TFH)), eight cases of peripheral T-cell lymphomas, not otherwise specified (PTCL-NOS), seven cases of anaplastic large-cell lymphomas, ALK-negative (ALCL, ALK-negative), three cases of intestinal T-NHL, two cases of NKTCL, two cases of T-cell large granular lymphocytic leukemia (T-LGL), and five cases of other subtypes (Mycosis fungoides (MF) *n* = 1, subcutaneous panniculitis-like T-cell lymphoma (SPTCL) *n* = 1, T-cell prolymphocytic leukemia (T-PLL) *n* = 1, cerebral T-NHL *n* = 1, and polymorphic post-transplant lymphoproliferative disorder (PTLD) *n* = 1). The recorded clinicopathological characteristics are summarized in Table 2. 

### 2.2. Sequencing and Mutation Analysis 

Whole exome sequencing (WES) was performed in a total of 11 MEITL and the available corresponding normal tissue from one tumor sample. Genomic DNA was extracted from FFPE tumor tissues and subsequently treated with uracil DNA glycosylase (UGD; GeneRead DNA FFPE Kit, Qiagen, Hilden, Germany) to suppress FFPE induced sequencing artifacts. After using the Agilent AllHuman V5 kit, sequencing was accomplished on an Illumina sequencing platform with paired end 100 base-pair reads. An FFPE-tissue optimized analysis pipeline was used for the analysis of single nucleotide polymorphisms (SNP) and copy number variations (CNV). SNP occurring in the sequenced normal tissue sample, dbSNP137, or in the 1000 genome database with a frequency of more than 1% were excluded. Mutations with allele frequencies below 10% were excluded because of the low frequency. MutSig and DOTS-Finder algorithms were used to identify the most significant and functional relevant mutations. 

Targeted sequencing was performed on the WES-cohort and 22 additional cases. A custom Haloplex panel (Agilent Technologies Inc., Santa Clara, CA, USA) targeting 71 genes, exomes, or hotspots of mutated T-cell neoplasms was used for mutational analysis. Libraries were sequenced using an Illumina system. Variants were called using the appreci8 pipeline [36]. Read depth was set to a minimum of 20, the minimum number of variant allele read to 5 and the minimum variant allele frequency (VAF) to 5%. The pipeline automatically filtered artifacts and SNPs based on call characteristics. These include, but are not limited to, a variant’s base quality, presence of a variant in common databases (e.g., dbSNP, 1000 genomes, and COSMIC) and the effect of a variant based on in silico prediction. Additionally, manual investigation of all borderline calls was performed using the integrated genome viewer (IGV).

### 2.3. Tissue Microarray Construction

TMAs were prepared using a manual tissue arrayer (Beecher Instruments Inc., Sun Prairie, WI, USA), as described previously [37]. Hematoxylin and eosin (H & E) staining was obtained from each donor block, and representative tumor regions were morphologically identified and marked by a pathologist (M.B. (Marcus Bauer)). Two 0.6-mm-diameter tissue cores were extracted from these marked areas and arranged on recipient paraffin blocks. Adequate controls for specific antibodies including liver tissue, tonsil tissue, breast cancer, seminoma, prostate carcinoma, and osteosarcoma were added. 

### 2.4. Immunohistochemistry and Scoring

Immunohistochemistry (IHC) for EZH1, EZH2, and H3K27me3 was performed following a standard protocol using a Bond III automated immunostainer (Leica Biosystems Nussloch GmbH, Wetzlar, Germany) and the Bond Polymer Refine Detection Kit (DS9800-CN). In addition to the tumor samples, 10 normal lymph node tissues were stained to compare the expression levels of healthy and tumor tissues. The primary antibodies used in this study were the following: EZH1 (1:100, Abcam, Cambridge, UK; ab137693), EZH2 (1:100; Cell Signaling, Danvers, MA; 3147s), and H3K27me3 (1:200, Cell Signaling, Danvers, 9733s). Immunostaining was assessed by two investigators (M.B. (Marcus Bauer) and F.L.S.) using the Zeiss Axioscope 5 microscope (Carl Zeiss Mikroskopie GmbH, Jena, Germany). The two investigators were blinded to pathologic and clinical data. Staining was evaluated semiquantitatively using the H-scoring method [38]. The H-score for one patient was calculated from the mean of two stains. In 89% of the cases, the results of the two examiners agreed, confirming the reproducibility of the used evaluation method. 

### 2.5. Statistical Analysis

Analyses were performed for the entire study cohort, followed by nodal T-NHL phenotypes. Comparison of continuous variables between two groups was evaluated with the Mann-Whitney *U* test and between multiple groups with the Kruskal–Wallis test using Bonferroni’s correction. Associations between the protein expressions were assessed using the Spearman correlation. Univariable overall survival (OS) and progression-free survival (PFS) analyses were performed with the Kaplan–Meier method. Statistical comparisons between groups were made by log rank tests. Multivariable analysis was performed using a Cox proportional hazards model with the Enter method to evaluate the impact of previously defined variables (age, sex, Ann Arbor stage, B symptoms, BMI, and protein expression of EZH1, EZH2, and H3K27me3) on PFS and OS. OS was defined as the time from primary diagnosis until last follow-up or death from any cause. PFS was defined as the time from primary diagnosis until lymphoma progression or death from any cause. Receiver operating characteristic (ROC) and Youden Index were used to determine a cutoff value for protein expression to divide the samples into two groups of high and low expression, respectively. Patients alive at the last follow-up date were censored. All *p*-values were interpreted exploratorily.

## 3. Results

### 3.1. EZH1 and EZH2 Mutations

**Figure 1:**
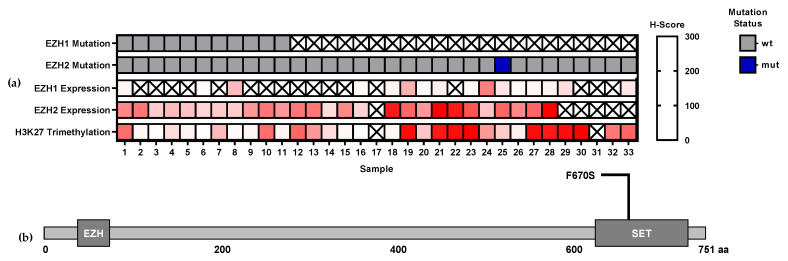
*EZH1* and *EZH2* mutations in monomorphic epitheliotropic intestinal T-cell lymphomas (MEITL): (**a**) *EZH1* and *EZH2* mutations and its association with EZH1, EZH2 and H3K27m3 protein expression. (**b**) Localization of the *EZH2* mutation in the SET-domain. Abbreviations: wt—wild type; mut—mutation.

In the MEITL cohort, the mutational status of *EZH1* and *EZH2* was investigated by next generation sequencing (NGS; Figure 1). *EZH1* mutation status was analyzed in 11 MEITL cases, but no mutation could be detected. *EZH2* mutation status was analyzed in 33 MEITL cases. A missense mutation with unknown biological impact located in the SET-domain (Figure 1b) was found to be present in one sample (3%). Immunohistochemical EZH1 (*n* = 16), EZH2 (*n* = 27), and H3k27me3 (*n* = 31) expression was also evaluated for the cases studied (Figure 1). Based on the one mutation found, no association with EZH1, EZH2, or H3K27me3 protein expression could be detected.

### 3.2. EZH1, EZH,2 and H3K27me3 Protein Expression Levels in T-Cell Lymphomas

**Figure 2:**
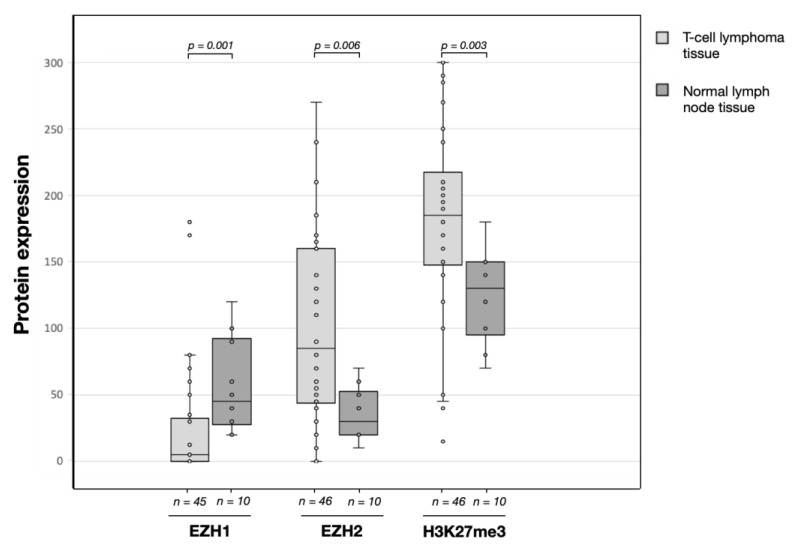
EZH1, EZH2, and H3K27me3 protein expression (H-score) in T-cell lymphomas and non-tumor tissues analyzed by immunohistochemistry. Boxplots represent the median and interquartile range of protein level (H-score). The number (*n*) of tissue samples is given below. *p*-values are shown above.

We compared the protein expression levels of T-cell lymphomas (*n* ≤ 46) with normal non tumor lymph node tissue samples (*n* = 10; Figure 2). EZH1 expression was found to be strongly decreased in tumor samples (median = 5; interquartile range (IQR) = 33) compared to normal lymphoid tissue (median = 45; IQR = 65; *p* = 0.001). In contrast, the expression of EZH2 (median = 85; IQR = 116) was increased compared to the controls (median = 30; IQR = 33; *p* = 0.016). Increased expression was also detected for H3K27me3 in tumor samples (median = 185; IQR = 70) compared to normal tissues (median = 130; IQR = 55; *p* = 0.054). 

**Figure 3:**
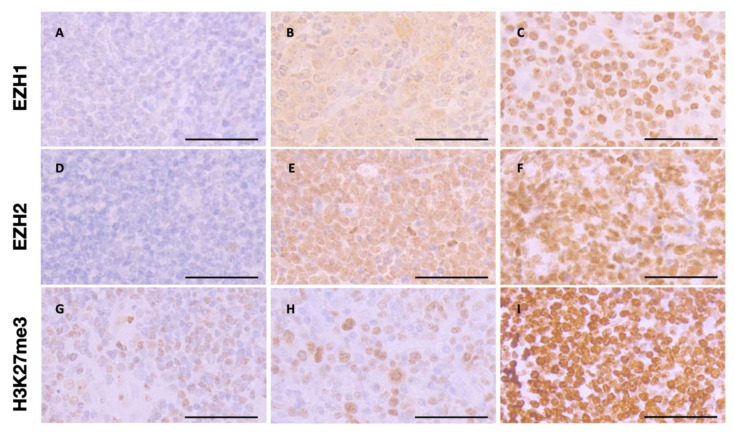
Representative immunohistochemical features of EZH1, EZH2, and H3K27me3. (**A**–**C**) Immunohistochemical staining showing low (**A**), middle (**B**), and high (**C**) levels of nuclear EZH1 expression. (**D**–**F**) Immunohistochemical staining showing low (**D**), middle (**E**), and high (**F**) levels of nuclear EZH2 expression. (**G**–**H**) Immunohistochemical staining showing low (**G**), middle (**H**), and high (**I**) levels of nuclear H3K27me3 expression. Original magnification ×400, the scale bars are 50 μm.

Representative immunohistochemical staining showing different levels of nuclear EZH1, EZH2, and H3K27me3 protein expression are illustrated in Figure 3.

### 3.3. Associations between EZH1, EZH2 and H3K27me3 Protein Expression

**Figure 4:**
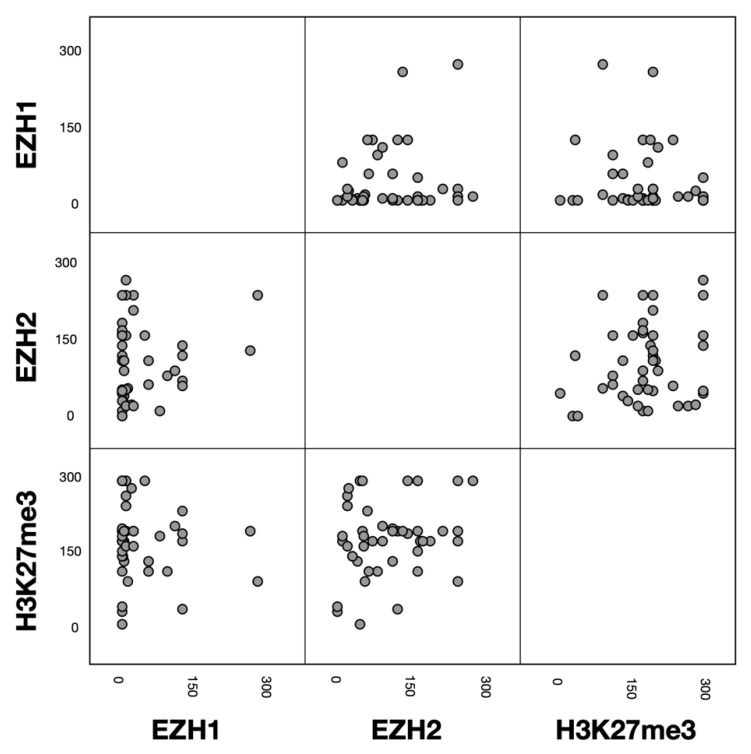
Scatterplot matrix visualizing the associations between EZH1, EZH2, and H3K27me3 immunohistochemical protein expression (H-score).

Correlations between EZH1, EZH2, and H3K27me3 protein expression (H-score) were analyzed in the entire study cohort. No correlation was observed between EZH1 and EZH2 (r = 0.127; *p* = 0.406; *n* = 45), EZH1 and H3K27me3 (r = 0.020; *p* = 0.899; *n* = 45), or EZH2 and H3K27me3 (r = 0.175; *p* = 0.224; *n* = 46). Visually, no other associations were detected between EZH1, EZH2, and H3K27me3 protein expression (Figure 4). 

### 3.4. Associations between EZH1, EZH2 and H3K27me3 Protein Expression and Clinicopathological Characteristics

**Figure 5:**
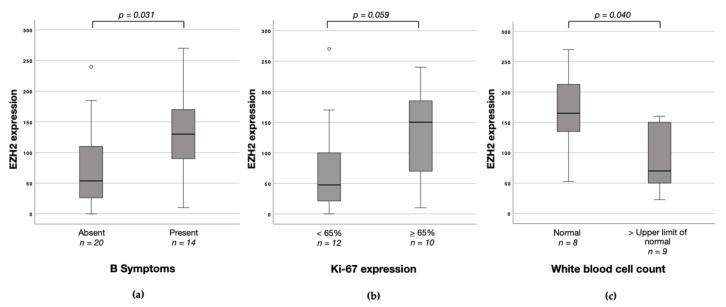
EZH2 protein expression (H-score) according to clinicopathological characteristics in nodal T-cell non-Hodgkin’s lymphomas (T-NHL). (**a**) Corresponding boxplot of EZH2 protein expression depending on the presence of B symptoms. (**b**) Corresponding boxplot of EZH2 protein expression depending on Ki-67 expression. (**c**) Corresponding boxplot of EZH2 protein expression depending on white blood cell count.

Possible associations between protein expression of EZH1, EZH2, and H3K27me3 with clinicopathological characteristics including sex, age, B symptoms, Ann Arbor stage, IPI, BMI, ECOG status, WBC, LDH, Ki-67 expression, the occurrence of relapses and response to first-line chemotherapy were investigated. No relevant associations were seen in the entire study cohort. In the nodal T-NHL cases, high EZH2 protein expression was associated with both the presence of B symptoms (B symptoms vs. no B symptoms: median 130 vs. 53.8; *p* = 0.031; Figure 5a) and with a high Ki-67 Index (Ki-67 Index > 65 vs. Ki-67 Index < 65: median 150 vs. 47.5; *p* = 0.059; Figure 5b). A normal white blood cell count (WBC) was also associated with high EZH2 protein expression (WBC normal vs. upper limit of normal: median 165 vs. 70; *p* = 0.040; Figure 5c). No associations with sex, age, Ann Arbor stage, IPI, BMI, ECOG status, LDH, the occurrence of relapses, and response to first-line chemotherapy were noted. Furthermore, no associations between EZH1 and H3K27me3 with clinicopathological characteristics were observed in nodal T-NHL.

### 3.5. Divergent Effects of EZH1 and EZH2/H3K27me3 Protein Expression on Patient Prognosis 

Survival analyses were performed separately for all markers in the two cohorts (entire study cohort and nodal T-NHL cohort). The cohorts were divided into two groups using ROC-analysis: Patients with high protein expression and patients with low protein expression. Using the 1-year landmark as the end point, the cutoff point was set at an H-score of 4 for EZH1, of 85 for EZH2, and of 203 for H3K27me3. At the time of analysis, median follow-up time for living patients was 25.0 months (range, 0 to 142). Overall, 21 patients (45.7%) had died.

**Figure 6:**
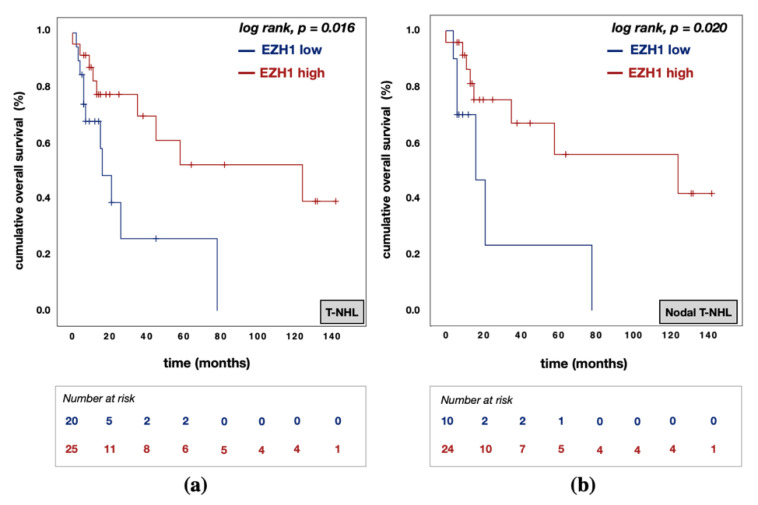
Kaplan–Meier (KM) curves for overall survival (OS) according to EZH1 protein expression. (**a**) KM curve for OS according to EZH1 protein expression in T-NHL; (**b**) KM curve for OS according to EZH1 protein expression in nodal T-NHL. Abbreviations: T-NHL —T-cell non-Hodgkin’s lymphomas; EZH1^low^—EZH1 protein expression with an H-score below 4; EZH1^high^—H3K27me3 protein expression above or equal to an H-score of 4.

In univariable analysis, EZH1^low^ expression was associated with poorer OS rates in the entire cohort (EZH1^low^ vs. EZH1^high^: median OS 16.0 (95% CI: 7.6–24.4) vs. 124.0 (95% CI: 16.4–232.0) months; *p* = 0.016; Figure 6a) and in nodal T-NHL cases (EZH1^low^ vs. EZH1^high^: median OS 16.0 (95% CI: 2.5–29.5) vs. 124.0 (95% CI: 0.0–272.9) months; *p* = 0.020; Figure 6b). However, EZH1 protein expression was not associated with PFS in univariable analysis.

**Figure 7:**
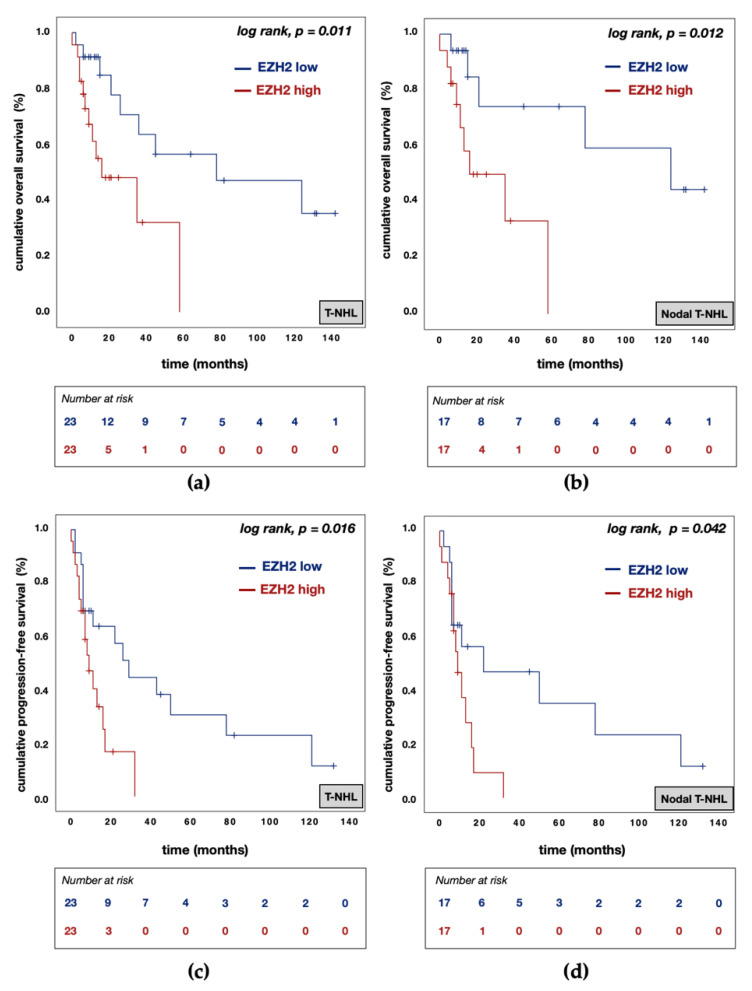
Kaplan–Meier (KM) curves for overall survival (OS) and progression-free survival (PFS) according to EZH2 protein expression. (**a**) KM curve for OS according to EZH2 protein expression in T-NHL; (**b**) KM curve for OS according to EZH2 protein expression in nodal T-NHL; (**c**) KM curve for PFS according to EZH2 protein expression in T-NHL; (**d**) KM curve for PFS according to EZH2 protein expression in nodal T-NHL. Abbreviations: T-NHL—T-cell non-Hodgkin’s lymphomas; EZH2^low^—EZH2 protein expression with an H-score below 85; EZH2^high^—EZH2 protein expression above or equal to an H-score of 85.

In contrast, EZH2^high^ expression was associated with poorer OS rates in the entire cohort (EZH2^low^ vs. EZH2^high^: median OS 78.0 (95% CI: 0.0–178.0) vs. 16.0 (95% CI: 0.0–38.5) months; *p* = 0.011; Figure 7a). This finding agrees with observations in the nodal T-NHL subtypes (EZH2^low^ vs. EZH2^high^: median OS 124.0 (95% CI: 13.8–234.2) vs. 16.0 (95% CI: 0.0–40.0) months; *p* = 0.012; Figure 7b). Furthermore, EZH2^high^ expression was associated with inferior PFS rates in the entire study cohort (EZH2^low^ vs. EZH2^high^: median PFS 29.0 (95% CI: 16.3–41.7) vs. 9.0 (95% CI: 4.2–13.8) months; *p* = 0.016; Figure 7c) and in nodal T-NHL cases (EZH2^low^ vs. EZH2^high^: median PFS 22.0 (95% CI: 0.0–71.0) vs. 9.0 (95% CI: 4.8–13.2) months; *p* = 0.042; Figure 7d). 

**Figure 8:**
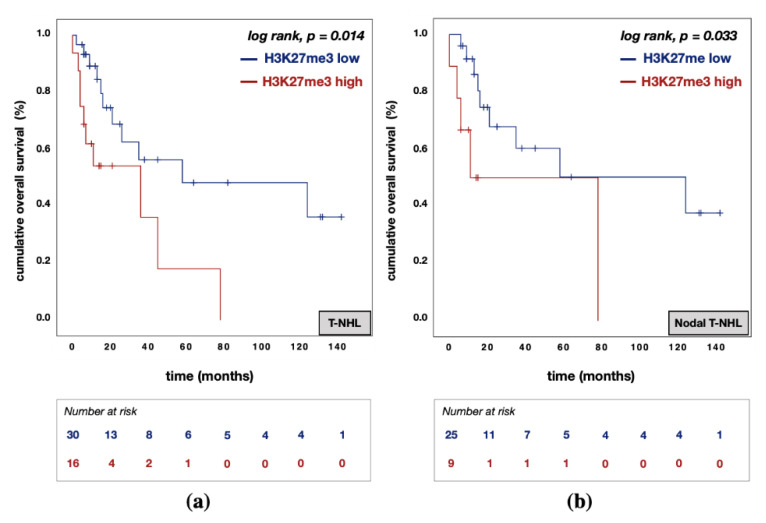
Kaplan–Meier (KM) curves for overall survival (OS) according to H3K27me3 protein expression. (**a**) KM curve for OS according to H3K27me3 protein expression in T-NHL. (**b**) KM curve for OS according to H3K27me3 protein expression in nodal T-NHL. Abbreviations: T-NHL—T-cell non-Hodgkin’s lymphomas; H3K27me3^low^—H3K27me3 protein expression with an H-score below 203; H3K27me3^high^—H3K27me3 protein expression above or equal to an H-score of 203.

Following the epigenetic modifiers EZH1/2 themselves, the influence of their consecutive histone lysine modification H3K27me3 on patient survival was investigated. In univariable analysis, H3K27me3^high^ expression was associated with poorer OS rates in the entire cohort (H3K27me3^low^ vs. H3K27me3^high^: median OS 58.0 (95% CI: 0.0–165.0) vs. 36.0 (95% CI: 0.0–73.7) months; *p* = 0.014; Figure 8a) and in nodal T-NHL cases (H3K27me3^low^ vs. H3K27me3^high^: median OS 124.0 (95% CI: 8.6–239.4) vs. 11.0 (95% CI: 0.0–50.4) months; *p* = 0.033; Figure 8b).

Multivariable Cox regression analysis of age, sex, Ann Arbor stage (only for nodal T-NHL), B symptoms, BMI, and protein expression of EZH1, EZH2, and H3K27me3 was performed for OS and PFS. This analysis revealed EZH1 expression (HR = 0.183; 95% CI: 0.044–0.767; *p* = 0.020; Table 3) and EZH2 expression (HR = 8.245; 95% CI: 1.898–35.826; *p* = 0.005; Table 3) to be independent prognostic markers for OS in the entire cohort. In nodal T-NHL cases, EZH1 (HR = 0.085; 95% CI: 0.008–0.859; *p* = 0.037; Table 3) and EZH2 (HR = 28.398; 95% CI: 2.166–372.334; *p* = 0.011; Table 3) were also independent prognostic markers for OS. 

In terms of PFS, BMI (T-NHL: HR = 4.621; 95% CI: 1.706–12.515; *p* = 0.003; nodal T-NHL: HR = 3.750; 95% CI: 1.252–11.231; *p* = 0.018; Table 3) and EZH2 expression (T-NHL: HR = 3.754; 95% CI: 1.233–11.426; *p* = 0.020; nodal T-NHL: HR = 5.147; 95% CI: 1.472–17.998; *p* = 0.010; Table 3) were independent prognostic markers in both cohorts. Age, sex, Ann Arbor stage, B symptoms, and H3K27me3 expression were not associated with OS or PFS in multivariable analysis.

## 4. Discussion

Aberrant epigenetic regulation has been shown to play a central role in the development of multiple malignancies. EZH2 functions as an important histone methyltransferase to regulate DNA methylation and control gene expression [12]. Over the past decade, studies have established that EZH2 is overexpressed in malignancies and that its high expression is associated with tumor progression and poor prognosis. In contrast, the function of EZH1 and expression status is a less studied field. In this study, we evaluated the *EZH1/2* mutation status in a cohort of 33 MEITL by NGS and investigated the immunohistochemical protein expression of EZH1, EZH2, and H3K27me3 in combination with the clinical outcomes in 46 patients with T-cell lymphomas. 

In NGS analysis, *EZH2* mutations were found to be present in just one MEITL case, which was located in the SET-domain. Furthermore, no *EZH1* mutation could be identified. On the basis of the single mutation found, we could not detect any associations with protein expression of EZH1, EZH2, or H3K27me3. Our results are in line with previous studies reporting that mutations of *EZH2* are rare in T-NHL: one in 36 cases (2.7%) in PTCL-NOS and one AITL case in 84 cases (1.2%) in a cohort consisting of AITL, PTCL-NOS, ALCL, and MEITL presented as nonsynonymous single nucleotide variants (SNV) [21]. In addition, several other groups have not identified mutations of *EZH1* by WES [17,18,19,20,21,22,23,24]. Mutations of other PCR2 complex members such as embryonic ectoderm development (*EED*) and suppressor of Zeste 12 (*SUZ12*) were not studied, but also not detected in other T-NHL studies [17,18,19,20,21,22,23,24].

Moreover, compared with normal lymph node tissues, EZH2 and H3K27me3 proteins were found to be overexpressed in T-cell lymphomas, whereas EZH1 was underexpressed, which is consistent with previous reports [25,26,27,28,29]. Our further findings, along with those of others [14,15,16,17] show that high EZH2 protein expression was associated with poor prognosis in T-cell lymphomas and markers that are known for cancer progression. Strong EZH2 protein expression was associated with the presence of B symptoms (median 130 vs. 53.8; *p* = 0.031) and high Ki-67 expression (median 150 vs. 47.5; *p* = 0.059) in nodal T-NHL. The univariable analysis showed poorer OS rates in both cohorts exhibiting high EZH2 protein expression compared with those exhibiting low expression (T-NHL: median OS 78.0 vs. 16.0 months; *p* = 0.011; nodal T-NHL: median OS 124.0 vs. 16.0 months; *p* = 0.012). Furthermore, high EZH2 protein expression was associated with inferior PFS rates (T-NHL: median PFS 29.0 vs. 9.0 months; *p* = 0.016; nodal T-NHL: median PFS 22.0 vs. 9.0 months; *p* = 0.042). In multivariable analysis, EZH2 was also an independent prognostic marker for OS (T-NHL: HR = 8.245; 95% CI: 1.898–35.826; *p* = 0.005; nodal T-NHL: HR = 28.398; 95% CI: 2.166–372.334; *p* = 0.011) and PFS (T-NHL: HR = 3.754; 95% CI: 1.233–11.426; *p* = 0.020; nodal T-NHL: HR = 5.147; 95% CI: 1.472–17.998; *p* = 0.010). 

H3K27me3 was also overexpressed and high protein expression also led to poor survival in univariable analysis (T-NHL: median OS 58.0 vs. 36.0 months; *p* = 0.014; nodal T-NHL: median OS 124.0 vs. 11.0 months; *p* = 0.033). However, H3K27me3 was not an independent prognostic marker in multivariable analysis and no associations with patient characteristics were observed. The correlation between EZH2 and its consecutive trimethylation of lysine 27 of histone H3 has previously been studied. In the context of T-cell lymphomas, results have been inconclusive so far: on one hand, a moderate positive correlation integrating multiple T-cell lymphoma entities (NKTCL, PTCL-NOS, AITL, ALCL, and T-LBL) was found [26], on the other hand, a strong inverse correlation of protein expression was reported in NKTCL [31]. Our analysis showed no correlation between EZH2 and H3K27me3 protein expression (r = 0.175; *p* = 0.224). Since there was no correlation between EZH2 and H3k27me3, no association between H3k27me3 and the studied clinicopathological markers, and the impact of H3K27me3 protein expression on OS could not be confirmed in multivariable analysis, we concluded that EZH2 may also influence the outcome of T-cell lymphomas by non-canonical functions.

Compared to the proteins already described, EZH1 was weakly expressed and associated with poorer survival when expressed at low levels. In univariable analysis, low EZH1 protein expression was associated with poor prognosis (T-NHL: median OS 16.0 vs. 124.0 months; *p* = 0.016; nodal T-NHL: median OS 16.0 vs. 124.0 months; *p* = 0.020). Furthermore, EZH1 protein expression was also an independent prognostic marker for OS (T-NHL: HR = 0.183; 95% CI: 0.044–0.767; *p* = 0.020; nodal T-NHL: HR = 0.085; 95% CI: 0.008–0.859; *p* = 0.037) in multivariable analysis. Thus, we can conclude that EZH1 and EZH2 have the opposite impact on the OS in the T-cell lymphomas cohorts studied. These results were replicated using The Cancer Genome Atlas (TCGA) (TCGA, https://tcga-data.nci.nih.gov/tcga/ (accessed on 29 October 2021)) with data from the TCGA Pan-Cancer (PANCAN) study and an inverse association to survival rates could also be observed (Supplementary Figure S1, available at Biomedicines online). Furthermore, Abdalkader et al. examined the association between EZH1 and EZH2 in T/NK-cell neoplasms and observed opposing protein expression patterns in both normal and neoplastic lymphoid tissues as well as an opposing relationship with Ki-67 expression [25]. Restrictively, it is to be added that our correlation analysis showed no association between the two paralogs EZH1 and EZH2, which could have been due to the extremely low or negative EZH1 expression.

Counterintuitively to our correlative study, in which EZH2 and EZH1 expression was not associated and low EZH1 expression conferred a poor prognosis, mechanistic studies revealed that dual inhibition of EZH1 and EZH2 is required for strong lethality in lymphomas due to increased EZH1 occupancy with focal H3K27me3 upregulation [12]. Valemetostat is a dual inhibitor of EZH1 and EZH2 that prevents trimethylation of H3K27, leading to altered gene expression patterns, which suppresses proliferation of EZH1/2-dependent cancer cells. Compared with established EZH2-specific inhibitors (GSK126), dual EZH1 and EZH2 inhibitors showed a significantly stronger reduction in H3K27me3 levels [12,39]. In an ongoing open-label phase 1 study (NCT 02732275, 2 November 2020 data cut-off) evaluating valemetostat tosylate monotherapy, the objective response rate (ORR) was 55.6% in 45 relapsed/refractory (r/r) PTCL patients and 50% in 14 r/r adult T-cell leukemia/lymphoma (ATL) patients [40]. The most frequently reported related treatment-emergent adverse events (TEAE) were decreased platelet count, dysgeusia, and anemia [40]. Based on these findings, a global phase 2 study of valemetostat tosylate monotherapy in patients with r/r peripheral T-cell lymphomas (n = 176) is currently ongoing (NCT 04703192).

Because of the retrospective nature of this analysis and the large heterogeneity of the studied cohort, the results have some limitations. The small sample size, which is unfortunately common in the study of T-cell lymphomas, carries the risk of statistical error. In addition, the extremely low protein expression of EZH1 posed a problem because it made staining evaluation difficult. Prospective studies with larger cohorts are therefore urgently needed to investigate the function of EZH1/EZH2 during carcinogenesis. In addition, further mechanistic experiments are required to elucidate the molecular mechanisms of EZH1 and EZH2 in T-cell lymphomas. 

In conclusion, we have demonstrated that *EZH1* and *EZH2* mutations are rare in MEITL and therefore do not appear to be of particular significance. Nevertheless, the proteins studied in T-cell lymphomas had opposite expression patterns and effects in survival analyses. In univariable analysis, high EZH1 protein expression was associated with an improved OS and PFS, whereas high EZH2 and H3K27me3 protein expression were associated with poorer OS and PFS. Multivariable analysis showed that EZH2 and EZH1 protein expression were independent, divergent prognostic markers for OS. EZH2 protein expression was also an independent prognostic marker for PFS. Targeting of both EZH1 and EZH2 enzyme activity may serve as a target for anticancer therapy in T-cell lymphomas.

## Figures and Tables

**Table 1:** Characteristics of mutation analysis patients.

Characteristics		MEITL *n* = 33
		*n* (%)
Sex	Female	16 (49)
	Male	12 (36)
	Not evaluable	5 (15)
Age	Median (years) (range)	62 (38–92)
Histopathology	CD3^positive^	33 (100)
	CD4^negative^	31(94)
	CD8^positive^	27 (82)
	CD58^positive^	31 (94)
T-cell Receptor	αβ^positive^	10 (30)
	γδ^positive^	9 (27)
	Not evaluable	14 (42)

Abbreviations: MEITL—monomorphic epitheliotropic intestinal T-cell lymphoma; *n*—number; CD—cluster of differentiation.

**Table 2:** Characteristics of tissue microarray patients.

Characteristics		T-NHL *n* = 46	Nodal T-NHL *n* = 34
		*n* (%)	*n* (%)
Sex	Female	15 (33)	12 (35)
	Male	31 (67)	22 (65)
Age	Median (years) (range)	64.1 (36–92)	64.4 (51–92)
B symptoms	Absent	27 (59)	20 (59)
	Present	18 (39)	14 (41)
	Not evaluable	1 (2)	0 (0)
Bone marrow involvement	Absent	32 (70)	25 (74)
	Present	10 (22)	7 (21)
	Not evaluable	4 (9)	2 (6)
Ann Arbor stage	Stages I and II	8 (17)	6 (18)
	Stages III and IV	28 (61)	25 (74)
	Not evaluable	10(22)	3 (9)
IPI	0–2	18 (39)	15 (44)
	3–5	19 (41)	17 (50)
	Not evaluable	9 (20)	2 (6)
ECOG	0–1	20 (43)	16 (47)
	2–5	6 (13)	5 (15)
	Not evaluable	20 (43)	13 (38)
WBC	Normal	13 (28)	8 (24)
	Upper limit of normal	11 (24)	7 (21)
	Not evaluable	22 (48)	19 (56)
LDH	Normal	6 (13)	2 (6)
	Upper limit of normal	19 (41)	17 (50)
	Not evaluable	21 (46)	15 (44)
Ki-67 expression	<65%	16 (35)	12 (35)
	≥65%	14 (30)	10 (29)
	Not evaluable	19 (35)	12 (35)
Relapse	Absent	23 (50)	14 (41)
	Present	23 (50)	20 (59)
First-line treatment	(R)-CHO(E)P	37 (80)	33 (97)
	Others	9 (20)	1 (1)

Abbreviations: T-NHL—T-cell non-Hodgkin’s lymphoma; IPI—International Prognostic Index, ECOG—Eastern Cooperative Oncology Group status; WBC—white blood cell count; LDH—lactate dehydrogenase level; (R)-CHO(E)P—(rituximab)-cyclophosphamide, doxorubicin, vincristine, (etoposide) and prednisone containing chemotherapy.

**Table 3:** Multivariable analysis of overall survival (OS) and progression-free survival (PFS).

		T-NHL (*n* = 41)				Nodal T-NHL (*n* = 30)			
Variable	Categories	HR	95% CI		*p*-Value	HR	95% CI		*p*-Value
			LL	UL			LL	UL	
**Overall Survival**									
Sex	female vs. male	3.816	0.990	14.699	0.052	2.900	0.462	18.198	0.256
Age in years		1.053	0.998	1.111	0.059	1.070	0.979	1.171	0.137
Ann Arbor stage	III–IV vs. I–II					0.148	0.021	1.042	0.055
B Symptoms	present vs. absent	1.182	0.411	3.397	0.756	1.066	0.166	6.822	0.947
Bone marrow involvement	present vs. absent	2.148	0.614	7.513	0.232	1.312	0.220	7.814	0.766
EZH1 expression	high vs. low	0.183	0.044	0.767	0.020	0.085	0.008	0.859	0.037
EZH2 expression	high vs. low	8.245	1.898	35.826	0.005	28.398	2.166	372.334	0.011
H3K27me3 expression	high vs. low	2.322	0.688	7.836	0.175	3.500	0.735	16.652	0.115
**Progression-Free Survival**									
Sex	female vs. male	1.607	0.630	4.097	0.321	0.959	0.289	3.179	0.945
Age in years		1.008	0.966	1.051	0.728	0.987	0.932	1.046	0.660
Ann Arbor stage	III–IV vs. I–II					0.933	0.249	3.495	0.918
B Symptoms	present vs. absent	1.299	0.512	3.298	0.582	0.888	0.250	3.159	0.855
Bone marrow involvement	present vs. absent	4.621	1.706	12.515	0.003	3.750	1.252	11.231	0.018
EZH1 expression	high vs. low	0.668	0.246	1.814	0.428	0.391	0.104	1.471	0.165
EZH2 expression	high vs. low	3.754	1.233	11.426	0.020	5.147	1.472	17.998	0.010
H3K27me3 expression	high vs. low	1.326	0.546	3.218	0.533	1.537	0.469	5.038	0.478

Abbreviations: T-NHL—T-cell non-Hodgkin’s lymphomas; HR—hazard ratio; CI—confidence interval; LL—lower limit; UL—upper limit; n—number; EZH1^high^—EZH1 protein expression above or equal to an H-score of 4; EZH1^low^—EZH1 protein expression with an H-score below 4; EZH2^high^—EZH2 protein expression above or equal to an H-score of 85; EZH2^low^—EZH2 protein expression with an H-score below 85; H3K27me3^high^—H3K27me3 protein expression above or equal to an H-score of 203; H3K27me3^low^—H3K27me3 protein expression with an H-score below 203.

## Data Availability

All relevant data are within the paper and its Supplementary Materials.

## Supplementary Materials

The following are available online at www.mdpi.com/article/10.3390/biomedicines9121842/s1, Figure S1: Kaplan–Meier (KM) curves for overall survival (OS) according to EZH1 and EZH2 protein expression in the TCGA Pan Cancer (PANCAN) study. (**a**) KM curve for OS according to EZH1 protein expression. (**b**) KM curve for OS according to EZH2 protein expression.

## References

[1] Swerdlow S.H., World Health Organization, International Agency for Research on Cancer (2017). WHO Classification of Tumours of Haematopoietic and Lymphoid Tissues.

[2] Vose J., Armitage J., Weisenburger D., International T.C.L.P. (2008). International peripheral T-cell and natural killer/T-cell lymphoma study: Pathology findings and clinical outcomes. J. Clin. Oncol..

[3] Bellei M., Federico M. (2019). The outcome of peripheral T-cell lymphoma patients failing first-line therapy: A report from the prospective International T-Cell Project. Haematologica.

[4] Czermin B., Melfi R., McCabe D., Seitz V., Imhof A., Pirrotta V. (2002). Drosophila Enhancer of Zeste/ESC Complexes Have a Histone H3 Methyltransferase Activity that Marks Chromosomal Polycomb Sites. Cell.

[5] Kuzmichev A., Nishioka K., Erdjument-Bromage H., Tempst P., Reinberg D. (2002). Histone methyltransferase activity associated with a human multiprotein complex containing the Enhancer of Zeste protein. Genes Dev..

[6] Müller J., Hart C., Francis N.J., Vargas M.L., Sengupta A., Wild B., Miller E.L., O’Connor M., Kingston R.E., Simon J.A. (2002). Histone Methyltransferase Activity of a Drosophila Polycomb Group Repressor Complex. Cell.

[7] Cao R., Wang L., Wang H., Xia L., Erdjument-Bromage H., Tempst P., Jones R.S., Zhang Y. (2002). Role of Histone H3 Lysine 27 Methylation in Polycomb-Group Silencing. Science.

[8] Barski A., Cuddapah S., Cui K., Roh T.-Y., Schones D.E., Wang Z., Wei G., Chepelev I., Zhao K. (2007). High-Resolution Profiling of Histone Methylations in the Human Genome. Cell.

[9] Margueron R., Reinberg D., Margueron R., Reinberg D. (2011). The Polycomb complex PRC2 and its mark in life. Nature.

[10] Shen X., Liu Y., Hsu Y.-J., Fujiwara Y., Kim J., Mao X., Yuan G.-C., Orkin S.H. (2008). EZH1 Mediates Methylation on Histone H3 Lysine 27 and Complements EZH2 in Maintaining Stem Cell Identity and Executing Pluripotency. Mol. Cell.

[11] Margueron R., Li G., Sarma K., Blais A., Zavadil J., Woodcock C.L., Dynlacht B.D., Reinberg D. (2008). Ezh1 and Ezh2 Maintain Repressive Chromatin through Different Mechanisms. Mol. Cell.

[12] Yamagishi M., Hori M., Fujikawa D., Ohsugi T., Honma D., Adachi N., Katano H., Hishima T., Kobayashi S., Nakano K. (2019). Targeting Excessive EZH1 and EZH2 Activities for Abnormal Histone Methylation and Transcription Network in Malignant Lymphomas. Cell Rep..

[13] Bachmann I.M., Halvorsen O.J., Collett K., Stefansson I.M., Straume O., Haukaas S.A., Salvesen H.B., Otte A.P., Akslen L.A. (2006). EZH_2_ expression is associated with high proliferation rate and aggressive tumor subgroups in cutaneous melanoma and cancers of the endometrium, prostate, and breast. J. Clin. Oncol..

[14] Duan R., Du W., Guo W. (2020). EZH2: A novel target for cancer treatment. J. Hematol. Oncol..

[15] Varambally S., Dhanasekaran S.M., Zhou M., Barrette T.R., Kumar C., Sanda M.G., Ghosh D., Pienta K., Sewalt R.G.A.B., Otte A.P. (2002). The polycomb group protein EZH2 is involved in progression of prostate cancer. Nat. Cell Biol..

[16] Wang C.-G., Ye Y.-J., Yuan J., Liu F.-F., Zhang H., Wang S. (2010). EZH2 and STAT6 expression profiles are correlated with colorectal cancer stage and prognosis. World J. Gastroenterol..

[17] Wang M., Zhang S., Chuang S.-S., Ashton-Key M., Ochoa E., Bolli N., Vassiliou G., Gao Z., Du M.-Q. (2017). Angioimmunoblastic T cell lymphoma: Novel molecular insights by mutation profiling. Oncotarget.

[18] Palomero T., Couronné L., Khiabanian H., Kim M.-Y., Ambesi-Impiombato A., Perez-Garcia A., Carpenter Z., Abate F., Allegretta M., Haydu J.E. (2014). Recurrent mutations in epigenetic regulators, RHOA and FYN kinase in peripheral T cell lymphomas. Nat. Genet..

[19] Sakata-Yanagimoto M., Enami T., Yoshida K., Shiraishi Y., Ishii R., Miyake Y., Muto H., Tsuyama N., Sato-Otsubo A., Okuno Y. (2014). Somatic RHOA mutation in angioimmunoblastic T cell lymphoma. Nat. Genet..

[20] Crescenzo R., Abate F., Lasorsa E., Gaudiano M., Chiesa N., Di Giacomo F., Spaccarotella E., Barbarossa L., Ercole E., Todaro M. (2015). Convergent mutations and kinase fusions lead to oncogenic STAT3 activation in anaplastic large cell lymphoma. Cancer Cell.

[21] Maura F., Dodero A., Carniti C., Bolli N., Magni M., Monti V., Cabras A., Leongamornlert D., Abascal F., Diamond B. (2021). *CDKN2A* deletion is a frequent event associated with poor outcome in patients with peripheral T-cell lymphoma not otherwise specified (PTCL-NOS). Haematologica.

[22] Roberti A., Dobay M.P., Bisig B., Vallois D., Boéchat C., Lanitis E., Bouchindhomme B., Parrens M.-C., Bossard C., Quintanilla-Martinez L. (2016). Type II enteropathy-associated T-cell lymphoma features a unique genomic profile with highly recurrent SETD2 alterations. Nat. Commun..

[23] Watatani Y., Sato Y., Miyoshi H., Sakamoto K., Nishida K., Gion Y., Nagata Y., Shiraishi Y., Chiba K., Tanaka H. (2019). Molecular heterogeneity in peripheral T-cell lymphoma, not otherwise specified revealed by comprehensive genetic profiling. Leukemia.

[24] Laginestra M.A., Cascione L., Motta G., Fuligni F., Agostinelli C., Rossi M., Sapienza M.R., Righi S., Broccoli A., Indio V. (2020). Whole exome sequencing reveals mutations in FAT1 tumor suppressor gene clinically impacting on peripheral T-cell lymphoma not otherwise specified. Mod. Pathol..

[25] Abdalkader L., Oka T., Takata K., Sato H., Murakami I., Otte A.P., Yoshino T. (2016). Aberrant differential expression of EZH1 and EZH2 in Polycomb repressive complex 2 among B- and T/NK-cell neoplasms. Pathology.

[26] Kim S.H., Yang W.I., Min Y.H., Ko Y.H., Yoon S.O. (2015). The role of the polycomb repressive complex pathway in T and NK cell lymphoma: Biological and prognostic implications. Tumor Biol..

[27] Yi S., Sun J., Qiu L., Fu W., Wang A., Liu X., Yang Y., Kadin M.E., Tu P., Wang Y. (2018). Dual Role of EZH2 in Cutaneous Anaplastic Large Cell Lymphoma: Promoting Tumor Cell Survival and Regulating Tumor Microenvironment. J. Investig. Dermatol..

[28] Yan J., Ng S.-B., Tay J.L.-S., Lin B., Koh T.L., Tan J., Selvarajan V., Liu S.-C., Bi C., Wang S. (2013). EZH2 overexpression in natural killer/T-cell lymphoma confers growth advantage independently of histone methyltransferase activity. Blood.

[29] Shi M., Shahsafaei A., Liu C., Yu H., Dorfman D.M. (2014). Enhancer of zeste homolog 2 is widely expressed in T-cell neoplasms, is associated with high proliferation rate and correlates with MYC and pSTAT3 expression in a subset of cases. Leuk. Lymphoma.

[30] Zhang H., Lv H., Jia X., Hu G., Kong L., Zhang T., Li L., Pan Y., Zhai Q., Meng B. (2019). Clinical significance of enhancer of zeste homolog 2 and histone deacetylases 1 and 2 expression in peripheral T-cell lymphoma. Oncol. Lett..

[31] Liu J., Liang L., Huang S., Nong L., Li D., Zhang B., Li T. (2019). Aberrant differential expression of EZH2 and H3K27me3 in extranodal NK/T-cell lymphoma, nasal type, is associated with disease progression and prognosis. Hum. Pathol..

[32] Cai M.-Y., Hou J.-H., Rao H.-L., Luo R.-Z., Li M., Pei X.-Q., Lin M.C., Guan X.-Y., Kung H.-F., Zeng Y.-X. (2010). High Expression of H3K27me3 in Human Hepatocellular Carcinomas Correlates Closely with Vascular Invasion and Predicts Worse Prognosis in Patients. Mol. Med..

[33] He L.-R., Liu M.-Z., Li B.-K., Rao H.-L., Liao Y.-J., Guan X.-Y., Zeng Y.-X., Xie D. (2009). Prognostic impact of H3K27me3 expression on locoregional progression after chemoradiotherapy in esophageal squamous cell carcinoma. BMC Cancer.

[34] Wei Y., Xia W., Zhang Z., Liu J., Wang H., Adsay V., Albarracin C., Yu D., Abbruzzese J.L., Mills G.B. (2008). Loss of trimethylation at lysine 27 of histone H3 is a predictor of poor outcome in breast, ovarian, and pancreatic cancers. Mol. Carcinog..

[35] Holm K., Grabau D., Lövgren K., Aradottir S., Gruvberger-Saal S., Howlin J., Saal L.H., Ethier S.P., Bendahl P.-O., Stål O. (2012). Global H3K27 trimethylation and EZH2 abundance in breast tumor subtypes. Mol. Oncol..

[36] Sandmann S., Karimi M., De Graaf A.O., Rohde C., Göllner S., Varghese J., Ernsting J., Walldin G., Van Der Reijden B.A., Müller-Tidow C. (2018). appreci8: A pipeline for precise variant calling integrating 8 tools. Bioinformatics.

[37] Schümann F.L., Bauer M., Groß E., Terziev D., Wienke A., Wickenhauser C., Binder M., Weber T. (2021). RBMX Protein Expression in T-Cell Lymphomas Predicts Chemotherapy Response and Prognosis. Cancers.

[38] Detre S., Jotti G.S., Dowsett M. (1995). A “quickscore” method for immunohistochemical semiquantitation: Validation for oestrogen receptor in breast carcinomas. J. Clin. Pathol..

[39] Kagiyama Y., Fujita S., Shima Y., Yamagata K., Katsumoto T., Nakagawa M., Honma D., Adachi N., Araki K., Kato A. (2021). CDKN1C-mediated growth inhibition by an EZH1/2 dual inhibitor overcomes resistance of mantle cell lymphoma to ibrutinib. Cancer Sci..

[40] Ishitsuka K., Izutsu K., Maruyama D., Makita S., Jacobsen E.D., Horwitz S., Kusumoto S., Allen P., Porcu P., Imaizumi Y. (2021). First-in-human Study of the EZH1 and EZH2 Dual Inhibitor Valemetostat Tosylate (Ds-3201b) in Patients with Relapsed or Refractory Non-Hodgkin Lymphomas. Hematol. Oncol..

