# Supplementary material for: Divergent Effects of EZH1 and EZH2 Protein Expression on the Prognosis of Patients with T-Cell Lymphomas
Source: Biomedicines. 2021 Dec 5;9(12):1842. doi: 10.3390/biomedicines9121842 (PMC8698684; doi:10.3390/biomedicines9121842)
Supplement: Supplementary file 1 [file biomedicines-09-01842-s001.zip › biomedicines-1465146-supplementary.pdf]

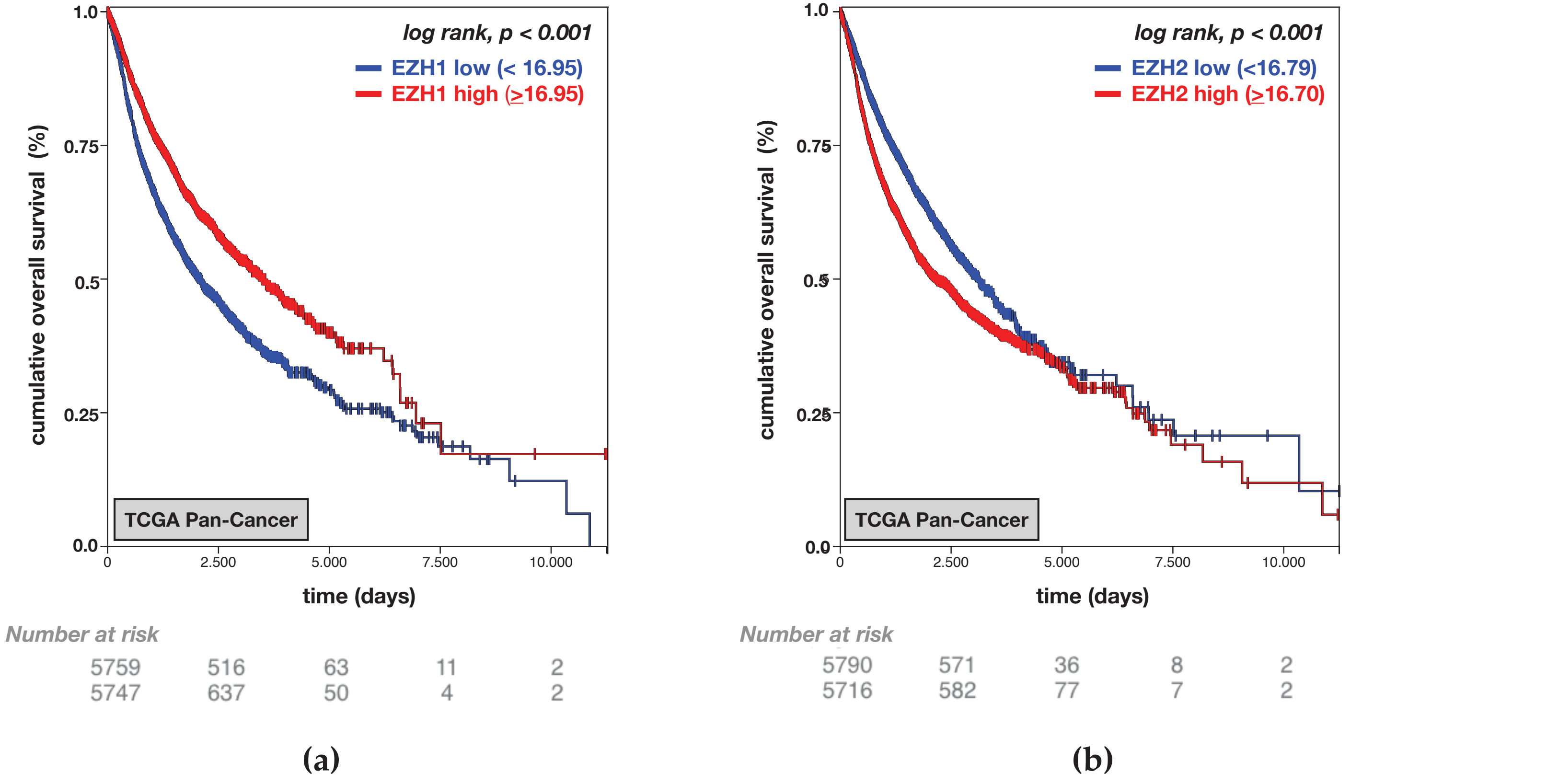

Figure S1: Kaplan–Meier (KM) curves for overall survival (OS) according to EZH1 and EZH2 protein expression in the TCGA Pan Cancer (PANCAN) study. (a) KM curve for OS according to EZH1 protein expression. (b) KM curve for OS according to EZH2 protein expression.
